# Hordes of Phages in the Gut of the Tilapia *Sarotherodon melanotheron*

**DOI:** 10.1038/s41598-018-29643-9

**Published:** 2018-07-27

**Authors:** Yvan Bettarel, Marine Combe, Antoinette Adingra, Awa Ndiaye, Thierry Bouvier, Jacques Panfili, Jean-Dominique Durand

**Affiliations:** 1 MARBEC, Univ. Montpellier, CNRS, Ifremer, IRD, Montpellier, France; 2 MIVEGEC, Univ. Montpellier, CNRS, IRD, Centre IRD de Cayenne, French Guiana, Cayenne, France; 3 Centre de Recherches Océanologiques, Abidjan, Ivory Coast France

## Abstract

Preliminary studies conducted on the human gastro-intestinal tract have revealed that enteric viral communities play a preponderant role in microbial homeostatis. However to date, such communities have never been investigated in the fish gut. Herein, we examined the main ecological traits of viruses in the digestive tract of a euryhaline fish, the tilapia S*arotherodon melanotheron*. Individuals were collected at 8 different sites in Senegal covering a salinity gradient from 3 to 104‰, and showing large disparities in their organic pollutant concentrations. Results showed that the gut of *S. melanotheron* is home to a highly abundant viral community (0.2–10.7 × 10^9^ viruses ml^−1^), distinct from the surrounding water, and essentially composed of phages of which a substantial proportion is temperate (the fraction of lysogenized cells-FLC ranging from 8.1 to 33.0%). Also, a positive and significant correlation was detected between FLC and the concentrations of polycyclic aromatic hydrocarbon in sediment, while no clear relationships were found between salinity and any of the microbial parameters considered. Finally, our data suggest that virus-bacteria interactions within the fish intestine are likely sensitive to the presence of particular xenobiotics, which may compromise the balance in the gut microbiota, and subsequently affect the health of their host.

## Introduction

The digestive tract of vertebrates houses an extraordinary number and variety of microorganisms including bacteria, viruses, fungi, and archaea which have co-evolved with their host, and are collectively referred to as the gut microbiota. Over the past two decades, researchers have tried to understand its implications for the host’s health, and its primary role in maintaining gut homeostasis. Commensal bacteria naturally present in the human intestinal tract are now well recognized to confer several beneficial functions to the host, not only for the digestion of food products but also for protecting from pathogenic microbial infections, and shaping the mucosal immune system^1,2^.

Most of the studies focusing on the gut microbiota of vertebrates have been conducted in humans, for which the medical challenge is of tremendous relevance^3^. However, little is known about the intestine microbiota of marine vertebrates, such as fish or mammals, despite their crucial ecological role and economical/societal values. The few information available suggests that the microbial network in fish gastrointestinal tract is very complex and probably plays a vital role in fish nutrition and disease prevention^4,5^. However, we still lack of clear information on how the microbial equilibrium is regulated within the intestine. Indeed, vertebrates need a stable, balanced gut bacteriome to be healthy^6^, and recently there has been increasing interest in understanding the factors that shape the activity and composition of enteric bacteria, as well as their subsequent role in host physiology and immunity.

Researchers discovered that viruses infecting bacteria (ie, phages) are highly abundant and diversified within the human gut, and forming key components of this specific microflora^7–9^. However to our knowledge, the occurrence, composition, and life traits of such enteric viruses have never been rarely explored in marine organisms for which they could also could play pivotal role, through lytic and/or lysogenic control of their bacterial hosts. For instance there is no information about viral abundances in the digestive tract of fishes, their infection strategies, nor their susceptibilty to ambient environmental conditions (salinity, temperature, xenobiotics, etc.). The only information available refers to the diversity of culturable phages in freshwater fishes (He & Yang 2015), which only represent a small fraction of the whole gastrointestinal community of viruses^10^.

In the present study, we examined the main ecological features of viruses present in the gut of the black-chinned tilapia (*Sarotherodon melanotheron*). This fish species was selected as a potential sentinel because of its abundance, physiological properties, and wide distribution in tropical coastal ecosystems. Indeed, this extreme euryhaline species, which is endemic to West Africa, is able to live in all types of water bodies, from freshwater to marine and even hypersaline waters^11^.

Individuals were collected from various sites in Senegal covering a salinity range from 3 to 104‰, and exhibiting contrasted concentrations of xenobiotics. This sampling strategy allowed us to (i) acquire basic information about the abundance, life strategies and morphological composition of viral communities found in the fish gut, and (ii) explore whether such viral communities are affected by salinity and/or the presence of persistent pollutants in their environment.

## Material and Methods

### Sampling sites

**Figure 1:**
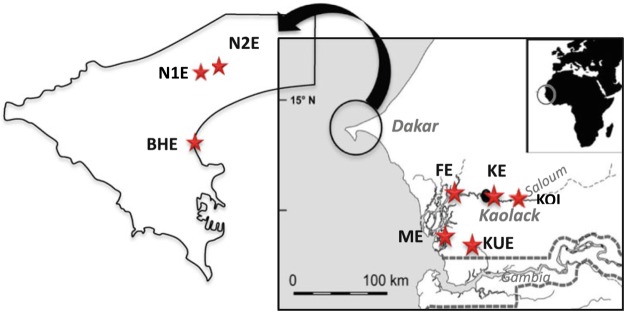
Sampling locations of the black-chinned tilapia *Sarotherodon melanotheron* in Senegal. Locations were characterized by salinities ranging from 3 to 104‰. BHE Hann Bay, FE Foundiougne, ME Missirah, N1E Niayes 1, N2E Niayes 2, KE Kaolack, KUE Koular, KOE, Koïlal.

The study was conducted in Senegal (West Africa) during the dry season (May 2010) at 8 contrasted stations, namely Niayes 1 (NE1), Niayes 2 (NE2), Koular (KUE), Kaoloack (KOE), Koïlal (KOI), Hann Bay (BHE), Foundiougne (FE), and Missirah (ME) (Fig. 1). These sites are located in the Dakar peninsula and along the estuary of Sine-Saloum, covering a salinity gradient from 3 to 104‰ (see Table 1).

**Table 1:** Water salinity and concentrations of the main persistent organic pollutants (in nanograms per gram dry weight of sediment) at the 8 sampling sites in Senegal (West Africa).

STATIONS	S (‰)	OCP (ng g^−1^)*	PCB (ng g^−1^)*	PAH (ng g^−1^)*
Niayes 2 (N2E)	2.7	32.5	0.6	30.7
Koular (KUE)	34.1	0.1	0.2	3.7
Hann Bay (BHE)	34.7	5.2	18.9	5855.6
Missirah (ME)	39.3	2.4	6.7	12.5
Foundiougne (FE)	51.4	0.5	3.8	14378.4
Niayes 1 (N1E)	58.6	1.4	0.6	23.7
Kaolack (KE)	102.0	10.4	1.0	62.3
Koïlal (KOI)	104.0	0.2	0.4	11.5

S, salinity; OCP, organochloride pesticides; PCB, polychlorinated biphenyls; PAH, polycyclic aromatic hydrocarbons (see *Ndiaye et al., 2012)*.

At each station, water salinity was measured with a refractometer. Surface sediment samples were also collected using a sediment corer (core tube 70 mm inner diameter; maximum sediment penetration depth of 6 cm), which allows collecting samples hermetically sealed. Visual inspection of the overlying waters and sediment surfaces revealed very limited resuspension effects during sampling. Sediment samples were then stored in aluminum containers and frozen at −20 °C for further chemical analysis of persistent organic pollutants such as polychlorinated biphenyls (PCB), organochlorine pesticides (OCP), and polycyclic aromatic hydrocarbons (PAH)^12^.

### Extraction of fish gut

At each sampling site, three adult tilapia *Sarotherodon melanotheron heudelotii* of similar size (130–150 mm fork length) were caught using a cast net and were anaesthetized progressively in icy water. Fish were then killed by cervical dislocation (following the European directive 2010/63/UE), as there is no regulation or guidelines in Senegal where experiments were carried out. Captured fish were individually placed in plastic bags and immediately stored in ice before dissection (within 5-h after sampling)^13^. Briefly, the digestive tract was extracted from each individual and cut from below the stomach to the rectum. Each gut was squeezed to expel content, which represented a minimum volume of 3 ml.

At each stations, triplicate raw gut contents (RGC) were processed for the different analyses as follows: (i) 1 ml was immediately processed for estimating the proportion of bacterial cells under lysogenic infections, and (ii) 2–3 ml were fixed with formaldehyde (final concentration f.c. 3% v/v), flash-frozen in liquid nitrogen and stored at −80 °C until measurement of viral and bacterial abundances, and distribution of the viral morphotypes. Triplicate subsurface water samples (20 ml) were also collected at each station, fixed with formaldehyde (f.c. 2% v/v) and stored at −80 °C in liquid nitrogen for determination of the parameters described above.

### Enumeration of viruses and prokaryotes

For each replicate, 1 mL of the fixed RGC was suspended in 1 mL of a 9‰ NaCl sterile saline solution complemented with 0.2 mL pyrophosphate (0.1 M) to extract bacterial and viral particles from gut matrix^13^. After vortexing for 2 min at room temperature, the supernatant was immediately transferred into a sterile tube containing 10 mL of 9‰ NaCl. The number of viruses and prokaryotes contained in each replicate samples was determined after retention of the particles on 0.02 µm pore-size membranes (Anodisc) and staining with SYBR Gold^14^. On each slide, 300–500 bacteria and viruses per replicate were counted with an Olympus Provis-AX70 epifluorescence microscope, under blue light excitation (488 nm). For planktonic viruses and bacteria, since the extraction procedure was not necessary, we only applied the staining protocol, as described above, by using a volume of 300–500 µL of seawater.

### Fraction of lysogenic bacteria

We used the method of Jiang and Paul^15^ to initiate prophage induction in gut and planktonic bacteria. Mitomycin C (final concentration: 1 mg mL^−1^, Sigma-Aldrich) was added to triplicate 1-mL volumes of unfixed and fresh RGC and water. Triplicate untreated samples served as control. All sub-samples were formalin fixed (f.c., 3% [vol/vol]) after being incubated for 12 h in the dark, at *in situ* temperatures (eg between 25.3–29.3 °C). Prophage induction was calculated as the difference in viral abundance (epifluorescence counts, see above) between mitomycin C-treated (Vm) and control incubations (Vc). The fraction of lysogenic bacterial cells (FLC) was calculated as follows:

$${\rm{F}}{\rm{L}}{\rm{C}}\,({\rm{ \% }})=100\times ({\rm{V}}{\rm{m}}-{\rm{V}}{\rm{c}})/{\rm{B}}{\rm{S}}\times {{\rm{B}}{\rm{A}}}_{{\rm{t}}0},$$

where BS is the burst size (number of viruses per bacterial cell) and BA_t0_ is the bacterial abundance at the start of the experiment, i.e., before adding mitomycin C^16^. Since no burst size from infected gut bacteria has been published so far, we used a burst size of 24, which is an average value calculated from the range for different studied marine environments^17^.

### Examination of viral morphotypes

The distribution of the different viral morphotypes in gut and water samples was examined using transmission electron microscopy (TEM)^18^. Viruses from 500 µL aliquots of formalin fixed and pyrophospate-treated samples (see above) were harvested by repeated ultracentrifugation of 50 µL onto grids (400 mesh Cu electron microscope grids with carbon coated Formvar film) using an A-100/30 rotor in an air-driven ultracentrifuge (Airfuge®, Beckman) at 105,000 × g for 70 min. The grids were then stained for 30 s with uranyl acetate (2%, w/w), and viruses were examined and measured using a JEOL 1200EX TEM operated at 80 kV and magnification from x20,000 to x100,000. Between 300 and 400 viruses were examined per grid. Three morphotypes were distinguished for shape classification of tailed viruses (*Caudovirales*). Tailed viruses with isometric heads and long non-contractile tails were considered to belong to the family *Siphoviridae*. Tailed viruses with isometric heads and contractile tails (presence of a neck) to the family *Myoviridae*. Tailed viruses with isometric heads and short tails to the *Podoviridae*. The proportion of tailless icosahedral viruses was also evaluated.

### Data analysis

Data were log transformed to satisfy the requirements of normality and homogeneity of variance necessary for parametric analyses. Simple relationships between original data sets were tested using Pearson correlation analysis. Statistical comparisons of mean values were performed using one-way Anova and Tukey post hoc tests. All statistical analyses were performed using SIGMASTAT software.

## Results and Discussion

The digestive tract of *S. melanotheron* harbored a highly concentrated community of viruses and bacteria, ranging respectively from 0.2 to 10.7 × 10^9^ viruses ml^−1^, and from 0.1 to 6.8 × 10^9^ bacterial cells ml^−1^ of gut content (Table 2). This is to our knowledge, the first report of viral abundances in the fish gut. Such abundances fall within the same order of magnitude of those commonly found in human gastrointestinal tracts (e.g., around 10^9^ viruses g^−1^)^8,19^, but are much greater than those reported in natural environments such as marine or even soil ecosystems, which rarely surpass 10^9^ viruses per milliliter of water or gram of soil^20,21^. Whilst the main information available on gut-associated viruses refers to those examined in humans^9,22,23^, the positive and highly significant correlation between viral and bacterial concentrations suggested that the fish gut viral communities were dominated by phages (Pearson correlation *r* = 0.90, p < 0.05, Table 3). This was also corroborated by the important proportion of tailed viruses of the Caudovirales families (*Sipho*-, *Myo*- and *Podoviridae*), which was significantly higher in the fish gut than in the surrounding water (Table 3). Given that the *Firmicutes*, *Proteobacteria* and *Bacteroidetes* typically represent up to 90% of the fish intestinal microbiota^4^, one may suspect that these abundant tailed and temperate phages are specific of these particular bacterial groups. Interestingly, similar bacteria prevalence were reported in the human gastrointestinal tract^1,24^ strongly supporting the paradigm of a core gut microbiome comprised of common bacterial members ensuring the primary functions of this vital organ (e.g., defense against pathogen, etc.). By analogy, one may also suspect the existence of a core viral diversity in vertebrate gut. The rest of the microbial community being rather dedicated to the specific digestion processes and diet, which are highly species-specific.

**Table 2:** Values (mean ± sd) for the different parameters measured in water samples (WAT) and digestive tracts (GUT) of *S. melanotheron*, in the 8 study sites in Senegal.

	STATIONS	BAC (10^9^ cells ml^−1^)	VIR (10^9^ VLP ml^−1^)	VBR	FLC (%)	SIPHO %	MYO %	PODO %	UNTAILED %
GUT	Niayes 2 (N2E)	2.2 ± 0.6	3.8 ± 0.3	1.7	19.3 ± 6.8	21.4	25.2	13.6	39.8
	Koular (KUE)	0.4 ± 0.1	0.4 ± 0.1	1.0	15.2 ± 4.1	13.8	22.1	33.1	31.0
	Hann Bay (BHE)	0.1 ± 0.1	0.5 ± 0.1	5.0	25.2 ± 9.8	18.2	2.2	6.5	73.1
	Missirah (ME)	1.0 ± 0.1	0.2 ± 0.1	0.2	18.2 ± 11.1	21.7	5.0	10.0	63.3
	Foundiougne (FE)	1.5 ± 0.3	2.7 ± 0.9	1.8	33.0 ± 8.9	38.3	11.5	32.8	17.4
	Niayes 1 (N1E)	6.8 ± 2.8	10.7 ± 3.2	1.6	8.1 ± 2.2	15.5	9.7	19.3	55.5
	Kaolack (KE)	5.3 ± 1.3	3.8 ± 0.9	0.7	11.2 ± 3.0	26.1	10.1	8.0	55.8
	Koïlal (KOI)	1.0 ± 0.4	1.7 ± 0.2	1.7	21.7 ± 4.0	23.0	2.4	6.0	68.6
	*MEAN ± SD*	2.3 ± 2.3^£^	3.0 ± 3.2^£^	1.5 ± 0.8^£^	19.0 ± 7.4^£^	22.2 ± 7.1^£^	11.0 ± 8.0	16.2 ± 10.5^£^	50.6 ± 18.2^£^
WAT	Niayes 2 (N2E)	1.9 ± 1.0	7.7 ± 1.7	4.1	0.7 ± 0.3	1.9	12.7	5.1	80.3
	Koular (KUE)	1.0 ± 0.1	3.8 ± 1.0	3.8	0.7 ± 0.1	16.2	17.2	5.4	61.2
	Hann Bay (BHE)	2.2 ± 0.3	4.9 ± 1.1	2.2	5.5 ± 2.0	15.0	7.3	9.1	68.6
	Missirah (ME)	0.7 ± 0.1	4.2 ± 1.1	6.0	0.3 ± 0.1	15.1	17.6	2.5	64.8
	Foundiougne (FE)	1.2 ± 0.1	7.1 ± 0.9	5.9	7.4 ± 0.5	9.0	16.1	4.8	70.1
	Niayes 1 (N1E)	2.1 ± 0.3	7.5 ± 1.2	3.6	0.9 ± 0.4	0.1	11.6	2.3	86.0
	Kaolack (KE)	3.4 ± 0.1	7.3 ± 2.0	2.1	4.3 ± 0.6	8.6	14.6	5.0	71.8
	Koïlal (KOI)	1.3 ± 0.3	4.4 ± 0.9	3.4	8.8 ± 1.9	8.7	11.6	2.9	76.8
	*MEAN ± SD*	1.7 ± 0.8	5.9 ± 1.6	3.9 ± 1.4	3.6 ± 3.2	9.3 ± 5.6	13.6 ± 3.3	4.6 ± 2.0	72.5 ± 7.7

BAC, bacterial concentrations; VIR, viral concentrations; FLC, fraction of lysogenized cells; PODO, proportion of Podoviridae; SIPHO, proportion of Siphoviridae; MYO, proportion of Myoviridae; UNTAILED, proportion of untailed viruses. ^£^indicates a significant difference in the mean values between water and gut samples (p < 0.05, one-way ANOVA, Tukey post hoc test).

**Table 3:** Pearson correlation coefficients between basic parameters in water (WAT) and digestive tracts (GUT) of *S. melanotheron*, in the 8 study stations.

		SAL	BAC	VIR	FLC	PODO	SIPHO	MYO	UNT	PCB	PAH	OCP	VBR
GUT	SAL	**1**	0.34	0.13	−0.19	−0.31	0.24	−0.56	0.33	−0.27	−0.11	−0.47	−0.20
	BAC		**1**	0.90**	−0.68	−0.07	−0.05	0.04	0.04	−0.43	−0.27	0.11	−0.27
	VIR			**1**	−0.53	0.08	−0.12	0.10	−0.04	−0.40	−0.15	0.10	−0.02
	FLC				**1**	0.18	0.66	−0.17	−0.29	0.43	0.83*	−0.08	0.48
	PODO					**1**	0.18	0.55	−0.89**	−0.34	0.44	−0.25	−0.10
	SIPHO						**1**	−0.13	−0.44	−0.06	0.74*	−0.02	−0.04
	MYO							**1**	−0.70	−0.52	−0.14	0.59	−0.20
	UNT								**1**	0.45	−0.49	−0.10	0.16
	PCB									**1**	0.36	−0.13	0.61
	PAH										**1**	−0.24	0.43
	OCP											**1**	0.05
	VBR												**1**
WAT	SAL	**1**	0.34	−0.03	0.59	−0.32	−0.04	−0.03	0.13	−0.47	−0.11	−0.2	−0.33
	BAC		**1**	0.61	0.09	0.32	−0.39	−0.44	0.39	0.03	−0.14	0.37	−0.77*
	VIR			**1**	−0.05	−0.07	−0.82*	−0.14	0.69	−0.29	0.20	0.54	−0.14
	FLC				**1**	0.19	0.09	−0.33	0.02	0.18	0.54	−0.32	−0.20
	PODO					**1**	0.40	−0.48	−0.36	0.70	0.36	0.19	−0.50
	SIPHO						**1**	0.25	−0.95**	0.50	0.13	−0.47	0.13
	MYO							**1**	−0.48	−0.53	−0.01	−0.16	0.66
	UNT								**1**	−0.33	−0.19	0.36	−0.24
	PCB									**1**	0.36	−0.13	−0.14
	PAH										**1**	−0.24	0.30
	OCP											**1**	−0.16
	VBR												**1**

BAC, bacterial concentrations; VIR, viral concentrations; FLC, fraction of lysogenized cells; PODO, proportion of Podoviridae; SIPHO, proportion of Siphoviridae; MYO, proportion of Myoviridae; UNT, proportion of untailed viruses; PCB, concentration of polychlorinated biphenyls; PAH, concentration of polycyclic aromatic hydrocarbons; OCP, concentration of organochloride pesticides; VBR, virus-to-bacteria ratio. Significant relationships are shown in bold (*p < 0.05; **p < 0.01).

The mitomycin-C inductions revealed a high proportion of lysogenic cells in the fish gut (min-max, 8.1–33.0%), which was significantly higher than that was found in their planktonic counterparts (min-max, 0.3–8.8%) (Table 2). However, incubations were performed *ex vivo* and therefore the results should be taken with caution. At first sight, such results are surprising as lysogeny is usually considered as a refuge strategy for viruses in habitats where the conditions for their reproduction is threatened by either low abundance and/or compromised host metabolism^25,26^. Yet, the gut of *S. melanotheron* contained a dense population of bacteria (eg, 0.1–6.8 × 10^9^ cells ml^−1^), resulting in a rather low virus-to-bacteria ratio (VBR) (m_gut_ = 1.5 ± 0.8) compared to that measured in the ambient water (mean_wat_ = 3.9 ± 1.4) (Table 2). These results clearly contrast with the refuge theory that predicts rather low prevalence of lysogeny in highly ‘bacterialized’ habitats, like the fish gut. Interestingly, such high proportions of lysogens were also found in the coral mucus which is a nutrient-rich medium, and which typically hosts an abundant community of bacteria^27,28^. Recently a new model, *the piggyback-the-winner model* postulates that lysogeny can dominate in certain biomes hosting abundant and fast growing bacteria^29^. This may be the case in animal microbiomes where commensal bacteria are abundant, functionally crucial for their host, and to which lysogeny could represent an advantageous strategy, providing cells immunity against viral surinfections together with acquisition of genes allowing optimal survival in stressing conditions^30,31^. Temperate phages have also been long shown to prevail in the human gut^9,32^, suggesting that the digestive tract may harbor a cosmopolitan lysogenic phageome capable of controlling the bacterial homeostastis in vertebrates at large. The metagenomic research of prophages sequences in a large variety of terrestrial and marine vertebrates will be necessary to further explore this sensitive question.

We did not observe any significant correlation between salinity and any of the studied microbial parameters. Overall, given the euryhaline nature of *S. melanotheron heudelotii*^12^ and because the fish intestine is a major osmoregulatory organ, it was not surprising to see that gut-associated bacterial and viral communities were not impacted by external osmolarity.

One of the most striking findings here was the positive correlation between the fraction of lysogenized cells and the concentrations of polycyclic aromatic hydrocarbons (PAH). We know that the gut bacteriome of fishes (and of human) is affected by external environmental conditions^33^, including the presence of xenobiotics^34,35^. However the potential relationships between gut-associated viruses and environmental pollutants have only received little attention so far. PAHs are ubiquitous environmental pollutants, generated primarily during the incomplete combustion of organic materials (e.g. coal, oil, petrol, and wood), which exhibit toxic, mutagenic and/or carcinogenic properties to many aquatic organisms^36,37^. Previous reports have shown that PAHs can accumulate into the sediment^38^, and since they are highly lipid soluble, they can be readily absorbed from the intestinal tract of marine vertebrates^39^. Herein, the positive relationship between the PAH concentrations and FLC may indicate that phage-bacteria interactions within the fish gut are also strongly susceptible to this specific persistent pollutants, contrary the other POPs investigated here (e.g. PCB and OCP). Since the lysis-lysogenic decision usually depends upon host cell metabolism^40^, one may suspect that the physiological stress caused by the presence of PAH could have triggered lysogenic pathways. This may impact the fragile balance between temperate and virulent phages within the gut microbiota, with potentially severe implications for fish health.
